# Molecular characterization of an endophytic *Phomopsisliquidambaris* CBR-15 from *Cryptolepis buchanani* Roem. and impact of culture media on biosynthesis of antimicrobial metabolites

**DOI:** 10.1007/s13205-014-0204-2

**Published:** 2014-03-19

**Authors:** H. C. Yashavantha Rao, Parthasarathy Santosh, Devaraju Rakshith, Sreedharamurthy Satish

**Affiliations:** 1 Department of Studies in Microbiology, University of Mysore, Manasagangotri, Mysore, 570 006 Karnataka India; 2 Plant Biotechnology Division, Unit of Central Coffee Research Institute, Coffee Board, Manasagangotri, Mysore, 570 006 Karnataka India

**Keywords:** Endophytic fungus, *Phomopsisliquidambaris*, *Cryptolepis buchanani*, Polyketide synthase gene, Antimicrobial metabolites

## Abstract

An endophytic fungus *Phomopsis liquidambaris* CBR-15, was isolated from *Cryptolepis buchanani* Roem. (Asclepiadaceae) and identified by its characteristic culture morphology and molecular analysis of the ITS region of rDNA and intervening 5.8S rRNA gene. The impact of different culture media on biosynthesis of antimicrobial metabolites was tested by disc diffusion assay. Polyketide synthase gene (PKS) of the endophytic fungus was investigated using three pairs of degenerate primers LC1–LC2c, LC3–LC5c and KS3–KS4c by PCR. TLC-bioautography method was employed to detect the antimicrobial metabolites. Antimicrobial metabolites fractionated with ethyl acetate extract showed significant antimicrobial activity against the test bacteria and fungi. Biosynthesis of antimicrobial metabolites was optimum as depicted by zone of inhibition from ethyl acetate extract cultured in potato dextrose broth. Strain CBR-15 was identified as *Phomopsisliquidambaris* and PKS genes of the fungus were amplified with LC3–LC5c and KS3–KS4c sets of degenerate primers. These findings suggest that endophytic *P.liquidambaris* CBR-15 harbor iterative type I fungal PKS gene domain which indicates the biosynthetic potential of endophytic fungi as producers of natural antimicrobial metabolites. The study also demonstrates the utilization and optimization of different culture media which best supports for the biosynthesis of the antimicrobial metabolites from *P.liquidambaris*.

**Electronic supplementary material:**

The online version of this article (doi:10.1007/s13205-014-0204-2) contains supplementary material, which is available to authorized users.

## Introduction

Natural products have been the major potential sources of chemical diversity while driving pharmaceutical discovery over the past century (Mishra and Tiwari 2011). Despite the present focus on synthetic products, natural products serve as continuing source of novel bioactive metabolites, retaining an immense impact on modern medicine (Wang et al. 2012). Microbial endophytes are viewed as an outstanding and unexplored source of novel bioactive natural products because many of them occupy literally millions of unique biological niches growing in a variety of unusual environments (Verma et al. 2007). Endophytic fungi are known to be as potential resources for producing bioactive compounds (Aly et al. 2010). They have proven to be promising sources of new and biologically active metabolites which are of interest for specific medicinal or agrochemical applications (Strobel and Daisy 2003). Infrequently, endophytic fungi capable of producing their host plant compounds have been discovered (Eyberger et al. 2006; Kusari et al. 2008, 2009a, b; Kusari and Spiteller 2011; Shweta et al. 2010). Based on knowledge of the chemistry and biology of endophytic fungi, the isolation of natural products can give us a platform to replace the existing synthetic drugs that provide resistance to pathogens and contaminate safe environment (Gond et al. 2012).

Polyketides have a great commercial interest for drug discovery and account for medicinal sales exceeding $20 billion per year (Cheng et al. 2009). They are large family of structurally diverse natural products found in plants, fungi and bacteria. The study of PKS gene in natural environments may provide important ecological insights, in addition to opportunities for antimicrobial drug development (Zhao et al. 2008). The production of antibiotics by filamentous fungi can be enhanced by genetic modification, mixed culture fermentation, immobilization of the cells, optimization of fermentation conditions or enzymes induction (Oyama and Kubota 1993; Ho et al. 2003). Even minor variations in the environment or nutrition have the potential to impact the quantity and diversity of fermentation products. As an initial step in media optimization, nutritional array could be applied to cognize the conditions in which they would be more apt to produce antibiotics or secondary metabolites, resulting in enriched biological activity (Bills et al. 2008).

*Cryptolepis buchanani* Roem. and Schult. belongs to the family Asclepiadaceae, a climbing tree which is widely used in folk medicine in Southeast Asia (Laupattarakasem et al. 2003). It also plays a great medicinal value in Ayurveda as anti-diarrheal, anti-inflammatory and blood purifier (Kaul et al. 2003). In view of this, *C. buchanani* was selected for the isolation of fungal endophytes.

Here, we report for the first time on incidence of endophytic fungus from *C. buchanani* Roem. which comprises KS domain of fungal PKS gene as indicators of bioactivity. The impact of different culture media on biosynthesis of antimicrobial metabolites from *P.liquidambaris* CBR-15 was evaluated. The endophytic fungus has been identified by molecular analysis of the ITS region of rDNA containing ITS1, ITS2 and the intervening 5.8S rRNA gene.

## Materials and methods

### Collection site and source of endophytic fungus

Mysore (12.3ºN 76.6ºE, elevation 754 m) is located in the Southern part of India which has an annual mean temperature of 30 °C with about 786 mm precipitation per annum. *C. buchanani* Roem. was selected for the present study from this region. The plant is located in the campus of Mysore University. Healthy asymptomatic leaf, bark and root samples were collected and brought to the laboratory in an icebox which was used to isolate endophytic fungus within 24 h of collection.

### Isolation of endophytic fungus

Collected samples were washed thoroughly in running tap water followed by distilled water before processing. To eliminate the epiphytic microorganisms, all the samples were initially rinsed with 70 % ethanol for 2 min and surface sterilized by sodium hypochlorite (4 %) for 5 min and again rinsed with 70 % ethanol for 30 s. The samples were rinsed two times in sterile double distilled water and allowed for surface drying in sterile conditions. The plant materials were cut into small segments (5 mm size) and placed on water agar plates (distilled water, 1.5 % agar) amended with chloramphenicol (250 ppm) and incubated at 30 °C for 3–4 days to few weeks till the growth initiated. The hyphal tips, that emerged from the plant tissues were picked and maintained on PDA plates for further studies (Wang et al. 2012). The endophytic fungus used in the present study is maintained in the Department of Studies in Microbiology, University of Mysore, India (Voucher number: MGMB/DCC/02/2012).

### Culture media

Four different culture media were used: Potato dextrose broth (PDB), Malt extract broth (MEB), Yeast extract sucrose broth (YSB) and Mycological broth (MCB).

### Fermentation and extraction

The endophytic fungus was cultured in 1-l Erlenmeyer flasks containing 500 ml of each different culture media for 3 weeks at 25 °C under static conditions. The culture broth was then filtered to separate the culture filtrate and mycelium. Culture filtrate was blended thoroughly and centrifuged at 4,000 rpm for 5 min. Liquid supernatant was extracted with an equal volume of ethyl acetate thrice separately and this was evaporated to dryness under reduced pressure at 45 °C using rotary flash evaporator (Buatong et al. 2011). All the experiments were conducted in triplicates.

### Test microorganisms

Gram-positive bacteria *Staphylococcusaureus* (MTCC 7443), *Bacillus subtilis* (MTCC 121), *Listeria monocytogenes* (MTCC 839). Gram-negative bacteria *Escherichia coli* (MTCC 7410), *Salmonella typhi* (MTCC 733), *Pseudomonas aeruginosa* (MTCC 7903), *Candida albicans* (MTCC 183) and *Fusarium verticillioides*.

### Antimicrobial susceptibility testing

The determination of antimicrobial susceptibility testing was carried out by disc diffusion assay. The sterile discs (5 mm) were impregnated with 20 μl (100 μg/disc) of ethyl acetate extracts obtained from different culture media. The discs impregnated with ethyl acetate extract were dried in laminar hood and placed on the surface of the media already seeded with test microorganisms in Petri plates. One control disc impregnated with only 20 μl of ethyl acetate was also placed for each test organism with a positive control. The plates were incubated at 37 ± 2 °C and room temperature (for test bacteria and fungi, respectively) and the diameter of the zone of inhibition was measured (Sadrati et al. 2013).

### Statistical analyses

Statistical analysis of results was performed using IBM SPSS version 20 (2011). One-way ANOVA (analysis of variance) at value *p* < 0.001 followed by Tukey’s Post Hoc test with *p* < 0.05 was used to determine the significant differences between the results obtained in each experiment.

### Molecular characterization of the strain CBR-15

#### Isolation of genomic DNA

The endophytic fungus was cultured in potato dextrose broth for 7 days at 30 °C under shaking conditions and the resultant mycelium was harvested by vacuum filtration and stored at −70 °C. The chilled mycelia were ground with mortar and pestle under liquid nitrogen then transferred into an Eppendorf microcentrifuge tube with 1 ml of pre-warmed (65 °C) 2 × CTAB extraction buffer (2 % w/v CTAB, 100 mM Tris–HCl, 1.4 M NaCl, 20 mM EDTA, 1 % β-mercaptoethanol, pH 8.0), and then incubated in a 65 °C water bath for 60 min with occasional gentle swirling. After centrifugation, the aqueous phase of the mixture containing the total DNA was reextracted with an equal volume phenol:chloroform:isoamyl alcohol (25:24:1). The residual phenol was removed with chloroform:isoamyl alcohol (24:1) twice. DNA in the aqueous phase was precipitated by adding 2 volume ethanol and 0.1 volume 3 M NaAc (pH 5.2) and then incubated at −20 °C overnight. The DNA pellet was washed with 70 % ethanol twice, and suspended in 50 μl of TE buffer (10 mM Tris–HCl, 1 mM EDTA, pH 8.0) (Kim et al. 2010).

#### PCR amplification of ITS region of rDNA

The ITS regions of the fungus were amplified by ITS primers, ITS1 (5′ TCCGTAGGTGAACCTGCGG 3′) and ITS4 (5′ TCCTCCGCTTATTGATATGC 3′) (White et al. 1990). The PCR amplification was carried out in 0.2 ml PCR tubes, using Master cycler personal (Eppendorf). The PCR reaction mixture (50 μl) contained 5 μl 10 × PCR buffer containing 15 mM MgCl_2_, 5 μl 2 mM deoxynucleoside triphosphates mix (dNTPs mix), 2 μl of each primer (5 pmol/μl), 4 μl template DNA, 2 μl (1 U/ml) *Taq* polymerase and deionised water (30 μl). Thermal cycling conditions were as follows: initial denaturation (4 min at 95 °C), followed by 30 cycles of denaturation (94 °C for 50 s), annealing (51 °C for 1 min), and primer extension (72 °C for 1 min), followed by final extension for 10 min at 72 °C. Amplification products were electrophoretically resolved on 1.4 % (w/v) agarose gel containing ethidium bromide (0.5 μg/ml), using 1 × TAE buffer at 70 V (Bhagat et al. 2012).

### Amplification of ketosynthase domain of fungal PKS gene of strain *Phomopsis iquidambaris* CBR-15

Three pairs of degenerate primers, LC1 and LC2c, LC3 and LC5c (Bingle et al. 1999), KS3 and KS4c (Nicholson et al. 2001), which are ketosynthase (KS) domain specific primers were used to amplify the KS domain sequence of the PKS genes of *P.liquidambaris* by PCR (Nicholson et al. 2001). PCR reactions (50 μl) contained approximately 4 μl genomic DNA template, 5 μl 10 × PCR buffer, 4 μl 2.5 mM of each dNTP, 3 μl of each primer, 1 μl of 2 U/μl *Taq* DNA polymerase and 30 μl deionised water. The thermal cycling program was as follows: 5 min at 94 °C; 34 cycles of 1 min at 94 °C, 1.5 min at 55 °C, 3 min at 72 °C and 10 min at 72 °C.

### TLC-bioautography assay

The antimicrobial activity of ethyl acetate extract was investigated by thin layer chromatography (TLC) using the bioautographic agar overlay method (Valgas et al. 2007). 10 μl of ethyl acetate extract cultured in each different media was spotted on precoated TLC silica gel plates (TLC, ALUGRAM^®^ SIL G/UV_254_, Machereye-Nagel, Germany) in an optimized solvent system of chloroform and methanol (9:1). The developed TLC plates were observed under visible light and UV light at 254 nm and 365 nm, respectively. The developed TLC plates were air dried and UV sterilized for 30 min. The TLC plates were then encased in sterile Petri plates and overlaid with Brain heart infusion medium (for *S.aureus*), Mueller–Hinton medium (for *E. coli*) and Sabouraud dextrose medium (for *C. albicans*) containing 0.65 % agar incorporated with 1 mg ml^−1^ 2,3,5-triphenyl tetrazolium chloride (Sigma-Aldrich) inoculated with 1 % standardized microbial inocula. After 8 h of diffusion at 8 °C, the plates were incubated for 24 h at 37 °C for bacteria and for 48–72 h at 25 °C for fungi, then for fungi the upper agar was sprayed with [3-(4,5 dimethylthiazol-2-yl)-2,5 diphenyltetrazolium bromide] (MTT) (Sigma-Aldrich) 5 mg mL–1 which was converted to a formazan dye by the test fungi. Inhibition zones were observed as clear spots against a red and purple background for bacteria and fungi, respectively. The areas of inhibition on the active spot were compared with the *R*_f_ value of the related spots on the reference TLC plate.

## Results and discussion

### Isolation of endophytic fungi and colony morphology endophytic fungal strain CBR-15

**Fig. 1:**
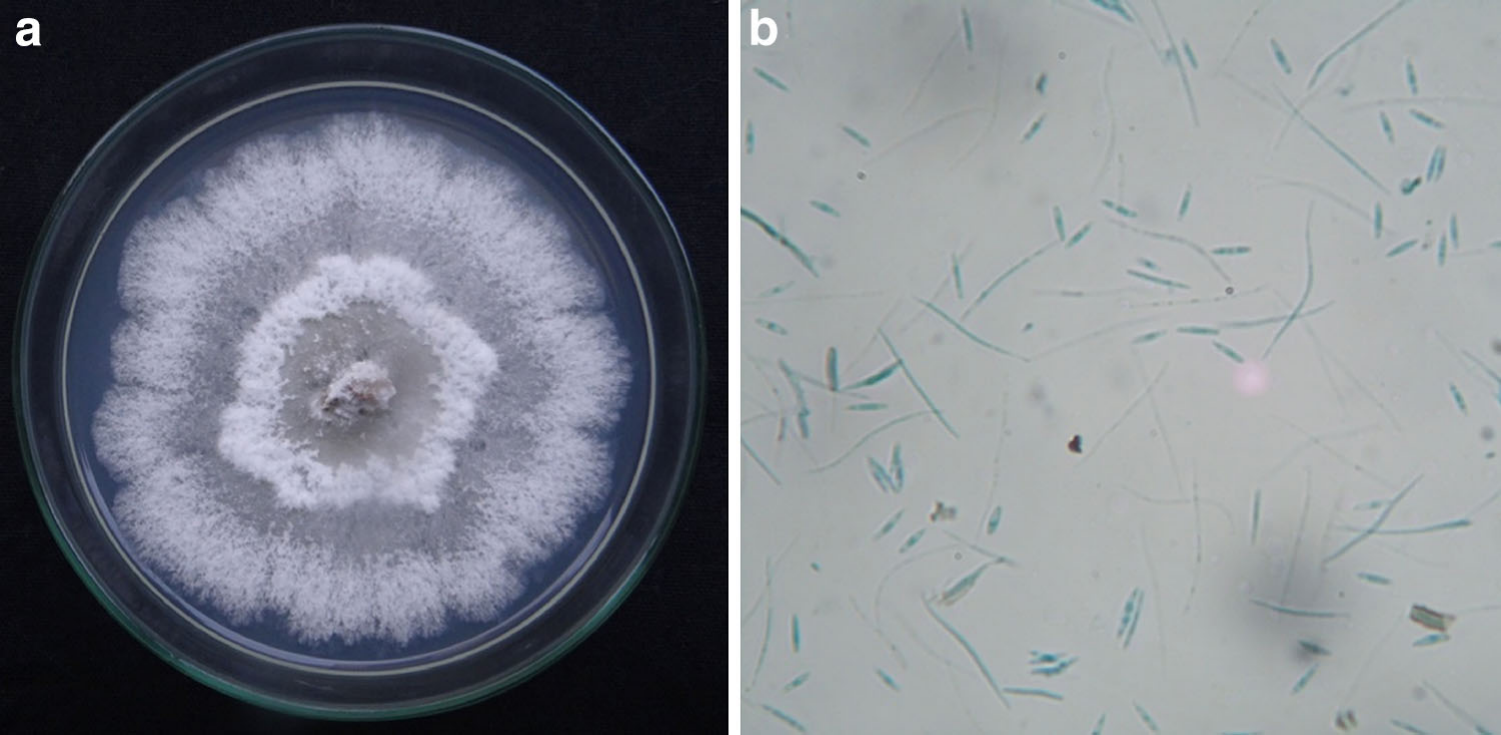
**a** Colony morphology of *P.liquidambaris* CBR-15 on PDA and **b** microscopic features at ×40 magnification showing alpha and beta conidia

The endophytic fungus *P.liquidambaris* CBR-15 which is used in the present study was isolated from the leaf tissue of *C. buchanani*. Colony morphology on PDA, after 5 days at 25 °C, initially cottony, white to olive gray later on turns to gray to light brown and a radiating growth pattern, margin regular. Conidiomata eustromatic, black, spherical to irregular on the upper regions; Conidiophores: short to elongated, aseptate to septate and branched, Conidia are of two types: (a) alpha conidia hyaline, straight, aseptate, forming white to yellowish cirrhi; (b) beta conidia hyaline, filiform, straight or curved, aseptae morphology characters observed, were closely related with the genus *Phomopsis*, belongs to class coelomycetes (Fig. 1).

### Impact of culture media on antimicrobial activity

Antimicrobial activity was determined by disc diffusion assay to assess the relative concentration of the active metabolites in ethyl acetate extract cultured in different media. The strain CBR-15 cultured on different media composition exhibited significantly distinct impacts on their antimicrobial activity. Different media compositions induced comparable antimicrobial responses in an individual culture media; however, minor variations were also observed. Interestingly, the optimal response in respect to antimicrobial activity was observed in the ethyl acetate extract cultured in PDB when compared to rest of the media (Table 1). This may be due to the need for certain nutritional supplements, which may serve as precursors, for the biosynthesis of bioactive secondary metabolites in endophytic fungi (Tong et al. 2011). Gram-positive bacteria were more susceptible than Gram-negative bacteria. Ethyl acetate extract of *P. liquidambaris* cultured in PDB exhibited zone of inhibition 22.33 ± 0.33 and 21.00 ± 0.00 mm for *S. aureus* and *E. coli*, respectively. Extract from YSB medium exhibited moderate antimicrobial response followed by PDB (Table 1).

**Table 1:** Antimicrobial activity of ethyl acetate extract of endophytic *P.liquidambaris* CBR-15 fermented in different culture media against test microorganisms by disc diffusion assay (100 μg/disc)

Culture media^A^	Test microorganisms							
	Bacteria						Fungi	
	Gram-positive			Gram-negative			Yeast	Mold
	*S. aureus*	*B. subtilis*	*Listeria monocytogenes*	*S. typhi*	*E. coli*	*P. aeruginosa*	*C. albicans*	*F. verticillioides*
PDB	22.33 ± 0.33^b^	24.66 ± 0.33^b^	22.00 ± 0.57	20.00 ± 0.00^b^	21.00 ± 0.00^a^	14.66 ± 0.33^b^	17.66 ± 0.66^b^	20.33 ± 0.33^a^
MPY	16.33 ± 0.33^e^	18.33 ± 0.33^d^	14.33 ± 0.33^e^	14.66 ± 0.33^d^	18.33 ± 0.33^d^	12.00 ± 0.57^c^	12.66 ± 0.33^d^	17.00 ± 0.57^b^
YSB	20.33 ± 0.33^c^	20.33 ± 0.33^c^	18.66 ± 0.33^c^	17.33 ± 0.33^c^	20.00 ± 0.57b	12.33 ± 0.33^c^	14.66 ± 0.33^c^	17.33 ± 0.66^b^
MCB	18.33 ± 0.33^d^	18.66 ± 0.33^d^	16.33 ± 0.33	15.00 ± 0.00d	16.66 ± 0.33^d^	13.66 ± 0.33^bc^	13.33 ± 0.33 ^cd^	16.33 ± 0.33^b^
Gentamicin (C)	28.00 ± 0.00^a^	33.33 ± 0.33^a^	24.33 ± 0.33^a^	30.33 ± 0.33^a^	29.66 ± 0.33^a^	20.33 ± 0.33^a^	ND	ND
Nystatin (C)	ND	ND	ND	ND	ND	ND	21.00 ± 0.00^a^	22.00 ± 0.00^a^

Value represents diameter of zone of inhibition in mm. Data are means from three replicates ± SE and those representing similar superscripts in the appropriate columns are not significantly different (ANOVA, Tukey’s HSD at *p* ≤ 0.05). C—positive control; Gentamicin—10 μg/disc, Nystatin—100 μg/disc

*ND* not determined

^A^See “Materials and methods” for abbreviation

**Fig. 2:**
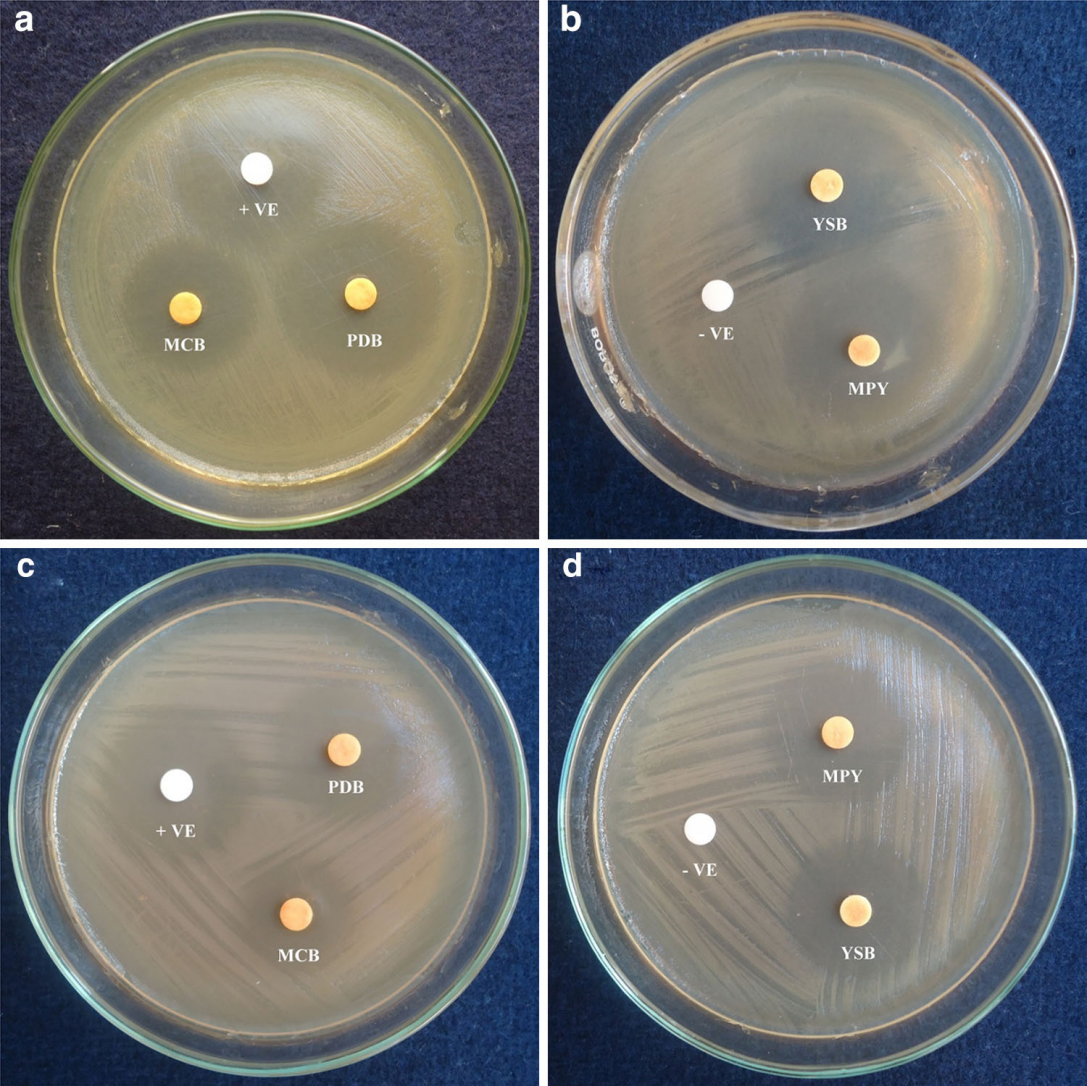
Antimicrobial activity of ethyl acetate extract of *P.liquidambaris* CBR-15 cultured in different media by disc diffusion assay against *E. coli* (*2a* and *2b*) and *B. subtilis* (*2c* and *2d*) where*+VE*  positive control,*−VE*  negative control and PDB,MPY, YSB, MCB are the different culture media (see “Materials and methods” for abbreviation) extract of *Phomopsisliquidambari* CBR-15

During disc diffusion assay, antimicrobial activity of ethyl acetate extracts cultured in different media were analyzed. The utilization of different mycological media as nutritional supplements can impact on the production of bioactive secondary metabolites. Application of multiple fermentation conditions is the desirable method that could enhance the probability of successful discovery of bioactive metabolites from a given strain (Bills et al. 2008). One such way to trigger the production of secondary metabolites is to vary the medium composition. The principle behind this method, named as one strain—many compounds (OSMAC) approach, is to expose the microorganism to other cultivating conditions than the standards used in laboratories (Fuchser and Zeeck 1997; Schiewe and Zeeck 1999; Hofs et al. 2000; Bills et al. 2008). Media composition, temperature, pH, culture vessel, aeration, cultivation time, light intensity can increase or reduce the production of the bioactive compounds by the strain (Bode et al. 2002; Siqueria et al. 2011). Yenn et al. (2012) reported anti-candidal activity of *Phomopsis* sp. ED2 cultured in yeast extract sucrose (YES) broth with aqueous extract of host plant. However, understanding of the exact mechanisms for the change in metabolic profile due to change in culture or fermentation conditions is usually not completely understood and therefore difficult to predict (Bode et al. 2002). This work demonstrates that PDB serves as optimum culture media for the biosynthesis of antimicrobial metabolites which facilitates isolation and characterization of antimicrobial metabolites from *P.liquidambaris* (Fig. 2).

### Molecular identification and amplification of ketosynthase domain of *Phomopsis* sp., PKS gene

**Fig. 3:**
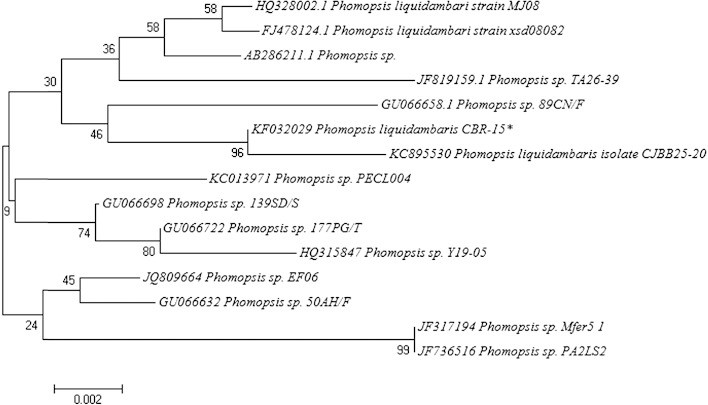
ITS sequence-based Neighbor Joining tree of *Phomopsis* sp. isolates. A consensus NJ dendrogram with bootstrap values (1,000 replications) based on multiple sequence alignment. *Scale bar* indicated nucleotide substitutions per nucleotide position. * denotes the isolate obtained in the present study (accession no. KF032029)

The amplified ITS region of rDNA was sequenced and aligned with the ITS sequences of the different organisms retrieved from NCBI databases, using CLUSTAL W (Thompson et al. 1994). Dendrogram was generated using neighbor joining (NJ) plot and the boot strapping was carried out using 1,000 replications. The acquisition of ITS1-5.8S-ITS2 sequence data and NJ plot showed that the isolate belongs to *P. liquidambaris* CJBB25-20, KC895530 (Fig. 3) which is also an endophytic fungus isolated from *Saraca asoca* (http://www.ncbi.nlm.gov/nuccore/KC895530). The partial ITS sequence data of this fungus was deposited in GenBank, under accession no. KF032029.

**Fig. 4:**
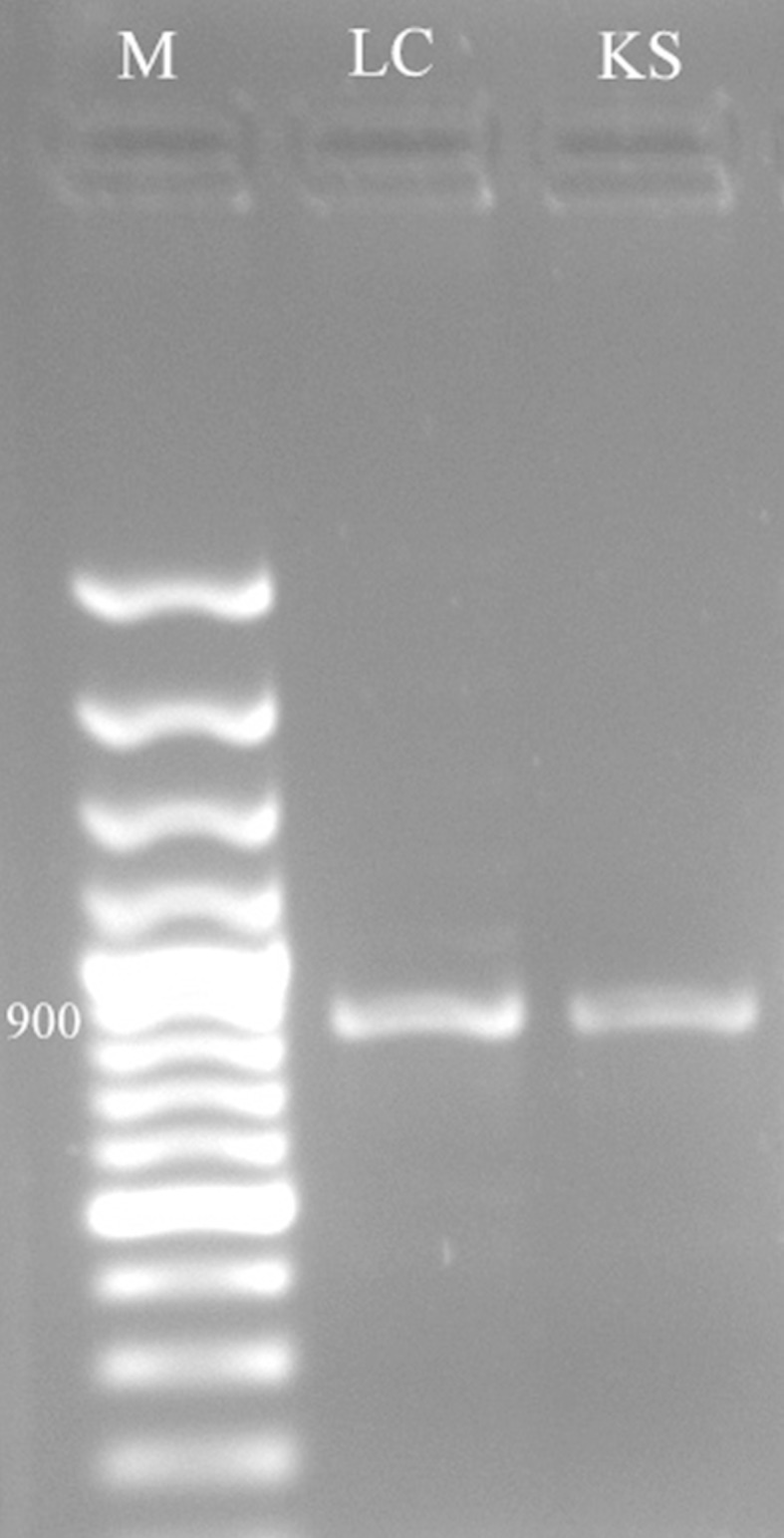
PCR amplification of polyketide synthase gene (amplicon size about 900 bp) from *P. liquidambaris* CBR-15 by LC3–LC5c and KS3–KS4c pairs of degenerate primers. Lane: *M* 100 bp DNA ladder; *LC* LC3–LC5c degenerate primers; *KS* KS3–KS4c degenerate primers

Traditional bioprospecting of microbial endophytes recently initiated a genetic-based screening program of culturable endophytes to identify strains capable of producing bioactive secondary metabolites. Endophytic fungi have been genetically screened for the presence of PKS genes as indicators of bioactivity. In the present investigation, the genomic DNA of *P. liquidambaris* CBR-15 was amplified by LC3–LC5c and KS3–KS4c sets of degenerate primers (Fig. 4). Fungal PKSs are defined as iterative type I synthases and are classified into three groups based on the extent of reduction of the polyketide ring produced, namely non-reduced, partially reduced and highly reduced PKSs (Nicholson et al. 2001). To identify the presence of iterative type I PKS in *P.liquidambaris* CBR-15, LC1–LC2c, LC3–LC5c and KS3–KS4c sets of degenerate primers were used which are specific for the particular types of the non-reduced, partially reduced and highly reduced KS domains of endophytic fungal PKS gene, respectively (Lin et al. 2010). From this study, *P.liquidambaris* CBR-15 might be capable of producing bioactive polyketide metabolites which are partially or highly reduced in nature due the amplification by LC3-LC5c and KS3-KS4c set of degenerate primers. The DNA of endophytic fungal strains isolated from *Annona squamosa* was investigated for PKS gene by KS domain specific primers. All three KS domains were present in the strains belonging to the Diaporthales (Lin et al. 2010). Investigation on PKS diversities in natural environment appears as an addition to opportunities for the development of microbial drugs which may provide important ecological insights (Zhao et al. 2008).

### TLC-bioautography

Metabolite profiling of ethyl acetate extract inclined from PDB medium by TLC showed better resolution of metabolites compared to rest of the media. Two major spots were observed under UV light of 254 and 365 nm, respectively. On TLC, no comparable good resolution of metabolites was observed other than cultured in PDB. This implies that the availability of nutrient supplements in PDB medium enhanced the production of bioactive metabolites of *P.liquidambaris* CBR-15. Certain nutrients act as environmental factors, quantitatively and qualitatively affecting the production of antimicrobial metabolites (Tabbene et al. 2009).

**Fig. 5:**
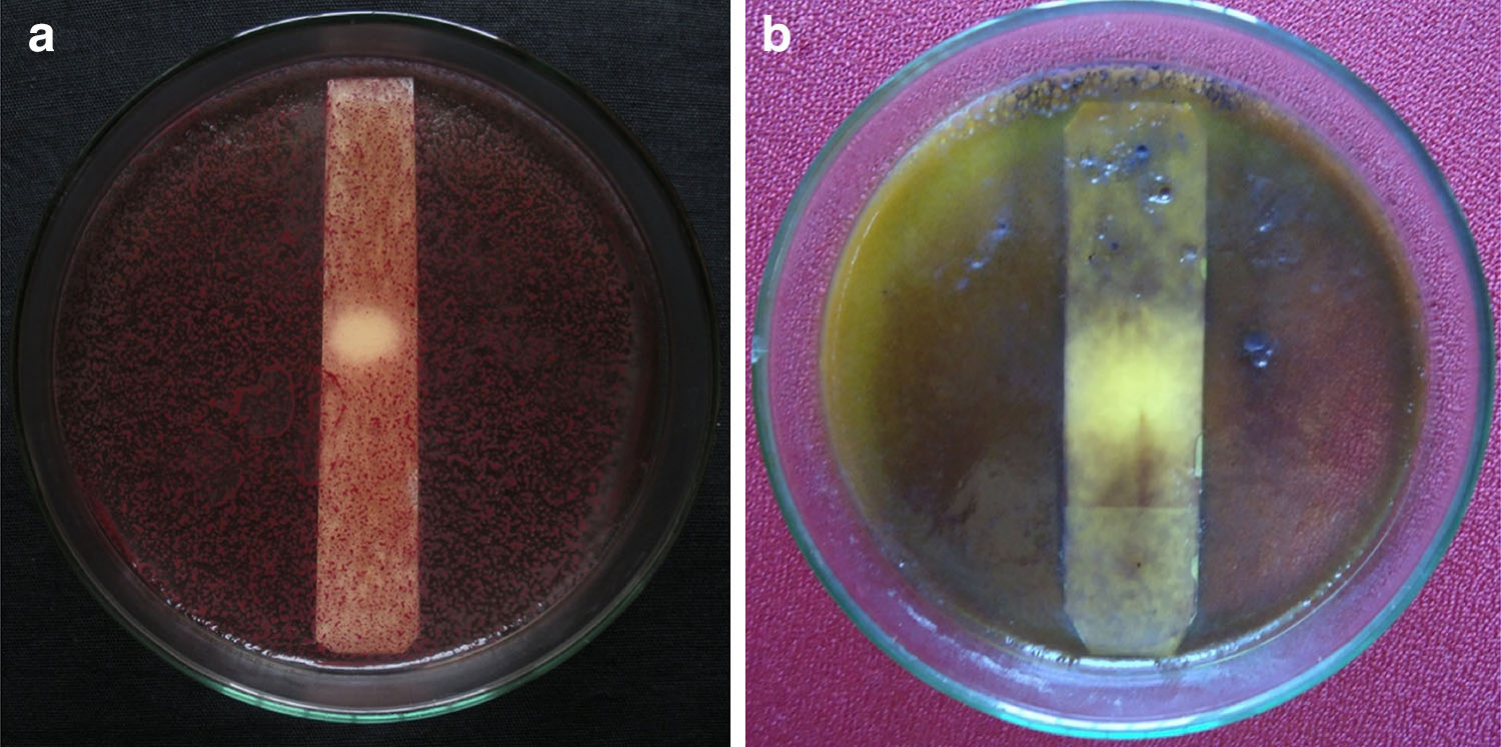
TLC-bioautography agar over lay assay of ethyl acetate extract cultured in PDB against **a***S. aureus* and **b***C. albicans*

In the bioautography assay, the ethyl acetate extract inclined from PDB culture filtrate exhibited antimicrobial activity by producing zone of inhibition at *R*_f_ value 0.56 against *S*. *aureus,E. coli and C*. *albicans* (Fig. 5). A mild activity was also observed from YSB culture at similar *R*_f_ value against *S*. *aureus and C*. *albicans*, but it did not show any activity against *E. coli*. This indicates that the compound against *E*. *coli* can only be produced when the *Phomopsis liquidambaris* strain CBR-15 is cultured in PDB. A spot with a similar *R*_f_ was not observed in the ethyl acetate extract from MPY and MCB media. Yenn et al. (2012) reported anti-candidal activity of *Phomopsis* sp. ED2 cultured in yeast extract sucrose broth with addition of host extract, but in our study we reported the antimicrobial activity of *Phomopsis* sp. CBR-15 without host extract. The present study for the detection of antimicrobial compound by TLC is one of the simplest, economical and reproducible methods for drug discovery from natural products (Ahmed 2008; Hota 2010; Marston 2011; Patra et al. 2012). Further investigation is needed to characterize the antimicrobial metabolite.

## Conclusions

Our finding implies that *P.liquidambaris* CBR-15, an endophytic fungus of *C. buchanani*, has antimicrobial properties and PDB is the best supporting media for the biosynthesis of antimicrobial metabolites. Bioactive natural compounds produced by endophytic fungi may provide new alternatives to address the problem of drug resistance development by human pathogens and multi-drug resistance microorganisms. The genome mining strategy employed here might assist strain prioritization for the isolation and characterization of antimicrobial metabolites with polyketide biosynthetic origin. This work is the first report on incidence of endophytic fungus inhabiting *C. buchanani* Roem. which comprises KS domain of fungal PKS gene as indicators of bioactivity.

## Electronic supplementary material

Below is the link to the electronic supplementary material.

Supplementary material 1 (DOCX 10 kb)
