# Supplementary material for: Molecular characterization of an endophytic Phomopsisliquidambaris CBR-15 from Cryptolepis buchanani Roem. and impact of culture media on biosynthesis of antimicrobial metabolites
Source: 3 Biotech. 2014 Mar 19;5(2):165–73. doi: 10.1007/s13205-014-0204-2 (PMC4362740; doi:10.1007/s13205-014-0204-2)
Supplement: Supplementary file 1 — Supplementary material 1 (DOCX 10 kb) [file 13205_2014_204_MOESM1_ESM.docx]

**Culture media**

Media were prepared by dissolving the ingredients in distilled de-ionized water. Potato dextrose broth (PDB): potatoes infusion 200 g/l, dextrose 20 g/l. Malt extract broth (MEB): malt extract 30 g/l, mycological peptone 5 g/l. Yeast extract sucrose broth (YSB): sucrose 30 g/l, sodium nitrate 3 g/l, potassium phosphate 1 g/l, yeast extract 1 g/l, potassium chloride 0.5 g/l, magnesium sulphate 0.5 g/l, ferrous sulphate 0.5 g/l. Mycological broth (MCB): papaic digest of soyabean meal 10 g/l, dextrose 40 g/l.
